# Minimally Invasive Redo Coronary Artery Bypass to Right Coronary Artery With Right Gastroepiploic Artery

**DOI:** 10.1016/j.atssr.2025.04.024

**Published:** 2025-05-17

**Authors:** Abel Cherian, Alexander Ryan, Yash Rohilla, Kevin Wang, Chidiebere Peter Echieh, Toshinobu Kazui

**Affiliations:** 1 College of Medicine–Tucson, University of Arizona, Tucson, Arizona; 2 College of Science, University of Arizona, Tucson, Arizona; 3 Department of Surgery, Banner–University Medical Center Tucson, Tucson, Arizona; 4 Division of Cardiothoracic Surgery, Department of Surgery, Banner–University Medical Center Tucson, Tucson, Arizona

## Abstract

Redo coronary artery bypass grafting (redo CABG) is a challenging procedure because surgical access must be reestablished, patent grafts must be preserved, and arterial graft selection is limited. We managed the case of a 58-year-old man with a history of prior CABG and end-stage renal disease in whom non–ST-segment elevation myocardial infarction developed as a result of severe in-stent restenosis of the mid right coronary artery. Coronary angiography demonstrated patent grafts, including left internal mammary to left anterior descending. To mitigate redo CABG risks, this patient underwent a sternotomy-sparing, off-pump, redo CABG by a gastroepiploic artery to posterior descending artery anastomosis through a minimally invasive subxiphoid approach.

Minimally invasive direct coronary artery bypass has been reported to spare the sternum to minimize surgical trauma and reduce patent graft injury, and it may be advantageous in redo surgical interventions.^1^ Relative to conventional redo sternotomy coronary artery bypass grafting (CABG), the potential benefits of a minimally invasive approach include avoidance of cardiopulmonary bypass and sternotomy, reduced stroke incidence, decreased blood transfusion requirements, mitigation of postoperative complications, and enhancement of various recovery metrics, including hospital length of stay and resumption of baseline activities.^2^ In addition, in redo operations in which conduit choices are limited because of prior use, the gastroepiploic artery (GEA) can be used as a bypass conduit as the clinical outcomes have been reported to be excellent and comparable to those of other conduits when used for bypassing the right coronary artery (RCA). ^1^^,^^3^ Last, when this conduit is used in a minimally invasive approach, the procedure may be potentially more beneficial in the setting of a patent left internal mammary artery (LIMA) to left anterior descending (LAD) graft as revascularization can be done without performing proximal anastomosis to the ascending aorta.

We experienced a case of redo CABG with a patent LIMA-LAD graft and recurrent in-stent RCA restenosis that underwent minimally invasive off-pump CABG with GEA through a subxiphoid approach and successful recovery from surgery.

A 58-year-old man presented to the emergency department with complaints of severe substernal chest pain that evolved into a non–ST-segment elevation myocardial infarction following a history of CABG with LIMA to the LAD anastomosis and saphenous vein graft (SVG) to the second obtuse marginal anastomosis 2.5 years ago. He also had 3 percutaneous coronary interventions (PCIs) on the RCA, with 2 stents inserted into the proximal and mid RCA about a year ago and a balloon angioplasty done in the mid RCA during this admission for immediate coronary perfusion. Additional past medical history includes dyslipidemia, hypertension, hypothyroidism, diabetes, and end-stage renal disease (ESRD) on peritoneal dialysis. Echocardiography demonstrated an ejection fraction of 60%. Coronary angiography demonstrated patent previous grafts (LIMA to LAD and SVG to second obtuse marginal artery), and the mid RCA was found to have diffuse in-stent restenosis with 99% occlusion and severe calcification that was successfully treated with balloon angioplasty (Figure 1).

**Figure 1:**
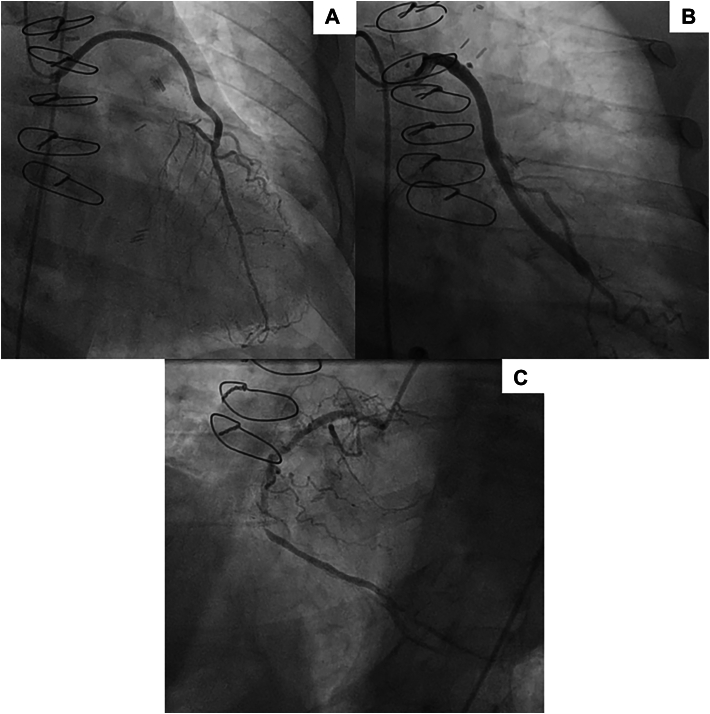
(a) Patent left internal mammary artery to left anterior descending artery anastomosed as a free graft. (b) Patent saphenous vein graft–second obtuse marginal artery as a free graft. (c) Diffusely diseased dominant right coronary artery with obstruction of the posterior descending artery.

We decided to proceed with a minimally invasive redo revascularization of the GEA to posterior descending artery (PDA). A 6-cm, downward midline skin incision was made from the xiphoid toward the abdomen, and the peritoneum was opened to expose the stomach. The GEA was identified and harvested from the stomach with a harmonic scalpel in a skeletonized fashion. The GEA was clipped at the distal end and placed in milrinone-containing saline syringes. The diaphragm was then opened down to the falciform ligament, and the pericardium was incised. The diaphragmatic surface of the heart was dissected. A self-retaining retractor was placed on the abdominal wound, and the coronary artery stabilizer was used to expose the PDA. An anastomosis of GEA to PDA was then performed in end-to-side fashion with 7-0 Prolene running suture (Figure 2). Because the PDA was heavily calcified, epicardial ultrasound was used to identify the optimal site for anastomosis and to ensure adequate coronary flow after anastomosis. Postoperative recovery was uneventful, and the patient was discharged on postoperative day 4.

**Figure 2:**
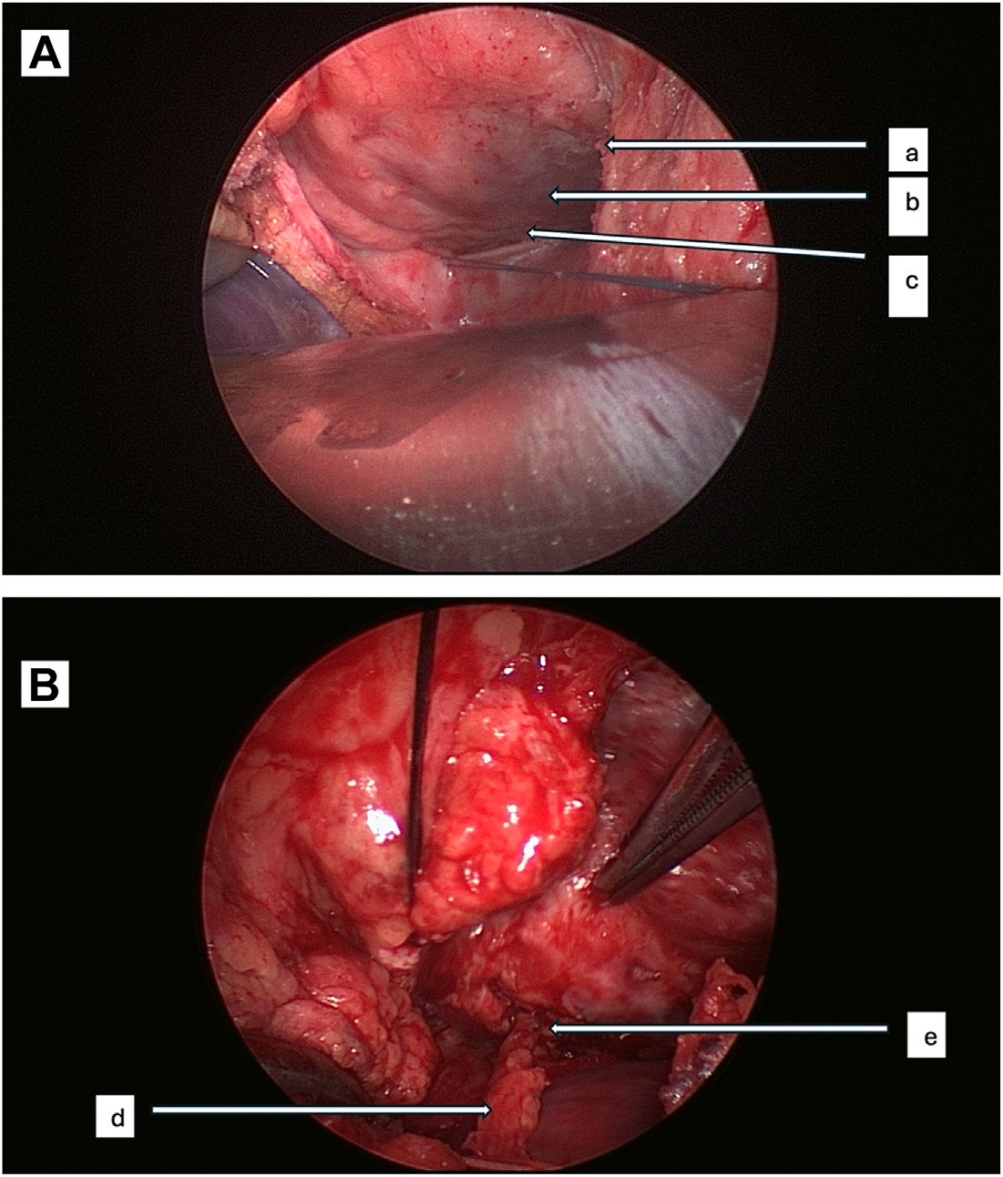
(A) Incision of the diaphragm to expose the inferior surface of the heart. (B) Anastomosis of the right gastroepiploic artery (GEA) to posterior descending artery (PDA). (a, divided diaphragm; b, diaphragmatic aspect of right ventricle; c, PDA; d, right GEA; e, right GEA-PDA anastomosis.)

## Comment

This was a unique case of a patient on peritoneal dialysis who successfully underwent minimally invasive redo CABG for PDA after 2 failed PCI stents.

An analysis of all isolated CABG procedures from 2002 to 2016 reported in the National Inpatient Sample database revealed that redo CABG procedures consisted of only 1.5% of the total procedures.^4^ This is in part due to redo CABG procedures being associated with higher in-hospital mortality secondary to operative complexity and adhesions, myocardial injury, cardiopulmonary bypass exacerbating underlying comorbidities, and patent graft injury.^5^ In fact, patients with patent LIMA-LAD from the primary procedure are generally excluded from redo CABG because of the risk of injury to the LIMA-LAD graft and subsequent development of myocardial infarction and death outweighing the benefit from non-LAD bypass.^6^ However, our relatively young patient was a good surgical candidate who could benefit more from redo CABG than from repeated PCI, given the history of failed previous PCIs to the RCA and active chest pain. In support of this argument, the most recent American Heart Association guidelines recommend that repeated revascularization should be pursued in cases in which patients have recurrent restenosis of prior stents and refractory angina.^2^ Thus, sternum-sparing, off-pump CABG using the GEA was the least risky but most effective option in this circumstance.

Regarding bypass conduit selection, the SVG is the most selected conduit in this case. However, the SVG requires proximal anastomosis to the ascending aorta, which requires dissection of the ascending aorta without injuring other bypass grafts. In addition, the long-term patency rate of venografts in patients with ESRD on hemodialysis is suboptimal.^7^ Another choice is the radial artery (RA), but it is not a good conduit of choice in this case because of potential use for dialysis access in the future and need for proximal anastomosis. With these constraints, the GEA became the best option as it is an in situ arterial graft. Although it is less frequently used compared with the internal mammary arteries and RA, good long-term outcomes have been reported and are comparable to those of the SVG and the RA when the target is a severely stenosed RCA.^3^ The GEA is a more muscular artery that is prone to spasm with surgical manipulation; however, use of antispasmodic agents can mitigate this challenge.^3^

The minimally invasive off-pump GEA to PDA approach can be a potential option for resolving myocardial ischemia in a young patient with patent grafts from prior CABG, RCA occlusion, and ESRD.
